# Diagnostic value of congenital pulmonary airway malformation volume ratio for fetal hydrops due to congenital lung malformations: a systematic review and meta-analysis

**DOI:** 10.1186/s13023-022-02347-0

**Published:** 2022-06-03

**Authors:** Pei Zhu, Kaisheng Cheng, Mingsheng He, Yutong Wang, Pengyue Shen, Kanglin He, Chang Xu, Ben Zhang, Zhenmi Liu

**Affiliations:** 1 Department of Epidemiology and Health Statistics, West China School of Public Health and West China Fourth Hospital, Sichuan University, Sichuan, China; 2 Department of Pediatric Surgery, West China Hospitial, Sichuan University, Sichuan, China

**Keywords:** CVR, Congenital lung malformation, Fetal hydrops, Systematic review, Meta-analysis

## Abstract

**Objective:**

Meta-analysis was used to evaluate the diagnostic value of a CVR cut-off value of 1.6 for fetal hydrops due to congenital lung malformation (CLM).

**Methods:**

A systematic search of PubMed, Embase, Web of Science, CNKI, VIP, and Wanfang published before 7/30/2021 for the value of a congenital pulmonary airway malformation volume ratio (CVR) cut-off value of 1.6 for the diagnosis of fetal hydrops. According to the inclusion and exclusion criteria, the literature that met the requirements were obtained. A total of 75 articles were retrieved, and 12 articles were included for further analysis. The quality of these studies was evaluated according to the Quality Assessment for Studies of Diagnostic Accuracy tool (QUADAS-2) criteria. The Q test and heterogeneity I^2^ were used to evaluate the heterogeneity due to non-threshold effects, and Stata 15.0 was used for statistical analysis to evaluate the diagnostic value of the CVR cutoff value of 1.6 for fetal hydrops due to CLM.

**Results:**

A total of 12 studies were included. The QUADAS-2 indicated that the risk of bias was relatively low, and the clinical applicability was relatively high. Statistical analysis was performed on included studies using a random effect model. Meta-analysis showed that the pooled sensitivity, specificity, diagnostic ratio and summary receiver operating characteristic (SROC) for the diagnosis of fetal hydrops by CVR were 0.86 (95% CI, 0.72–0.93; *I*^2^ = 59.84), 0.90 (95% CI, 0.88–0.93; *I*^2^ = 31.94), 58 (95% CI, 22–149; *I*^2^ = 100%), 0.93 (95% CI, 0.91–0.95).

**Conclusions:**

The sensitivity and specificity of CVR cut-off value 1.6 for the diagnosis of CLM-induced fetal hydrops were high, no publication bias was observed, and the CVR cut-off value 1.6 is meaningful for the early diagnosis prediction of CLM-induced fetal hydrops.

## Introduction

CLM is a collective term for a range of disorders that include the congenital abnormalities of the lung parenchyma and its bronchovascular structures. Among these malformations, congenital pulmonary airway malformation (CPAM), pulmonary sequestration (PS), and congenital lobar emphysema (CLE) are more common [1, 2]. CPAM is a nonfunctioning multicystic congenital pulmonary tissue dysarthria, with histological features of terminal bronchiolar overgrowth and the absence of normal alveoli [3]. PS is a non-functional lung mass, which is separated from the normal trachea and bronchus and receives blood supply from systemic circulation [4]. These masses may obstruct venous return and complications such as fetal hydrops, which can cause fetal death in severe cases. Studies have shown that the survival rate of the fetus with edema is only 3% [5], and is associated with several obstetric complications, such as preterm delivery [6] and high stillbirth rate [7]. Therefore, carrying out the prediction and early diagnosis of the risk of developing fetal hydrops caused by CLM and implementing preventive interventions and treatment measures for high-risk groups are essential to reduce the incidence of fetal hydrops and improve the quality of prognosis of the child.

CVR is a index for predicting fetal hydrops. It can be used to determine the volume ratio of the lesion to the lung, describe the relationship between the lesion and the lung, and predict fetal edema and prognosis. Calculation of CVR, the length, width, and depth of the mass were multiplied by a 0.52 correction factor (with the assumption that its shape was a prolate ellipse) and then divided by the head circumference to normalize for gestational age [8]. CVR is widely used in clinical practice because it can predict and judge the lesion size and gestational week together. Various cutoff values have been reported for CVR, such as 2.0 [9], 1.68 [10], 1.26 [11], and 1.6 [9].

One study has shown that the diagnostic sensitivity and specificity of fetal hydrops reached 93% and 63%, respectively, using 1.6 as the cut-off value [8]. In the later period, the diagnostic value of the CVR cut-off value of 1.6 was verified by other studies [12–15]. However, the results of the recent studies are inconsistent due to the differences in ethnics and sample sizes. Therefore, the CVR cut-off value of 1.6 for clinical guidance needs to be further validated [9, 16].

This study proposes to conduct a systematic review and Meta-analysis to evaluate the effects and the quality of evidence for the diagnostic value of CVR cut-off value 1.6 for fetal hydrops caused by CLM.

## Materials and methods

### Literature search strategy

The literature published by PubMed, Embase, Web of Science, CNKI, VIP, and Wanfang before July 30, 2021, evaluated the CVR cut-off value of 1.6 in diagnosing fetal hydrops caused by CLM, were systematically searched. Listed references were also manually checked for relevant papers. The search terms used in combination: (1) congenital lung cystic malformation, congenital pulmonary cystic lesions, congenital cystic adenomatoid malformation of lung, congenital pulmonary airway malformation; (2) cvr, cpam-volume ratio, congenital pulmonary airway malformation-volume ratio; and (3) sensitivity, specificity, diagnostic test; roc; diagnostic accuracy.

### Inclusion and exclusion criteria

*Inclusion criteria* The study population was fetuses diagnosed with CLM by prenatal ultrasound. The study reported fetal ultrasound findings including fetal hydrops. Fetal hydrops: It was defined as the presence of fluid in ≥ 2 spaces, which included ascites, pleural effusion, pericardial effusion, or skin edema [17], which has been considered the standard obstetric definition of this disorder. Fetal hydrops was assessed using a CVR cutoff value of 1.6. Exclusion criteria: repeat literature; "Studies not provided sufficient data for calculating the true positive (TP), false positive (FP), false negative (FN) and true negative (TN) values; reviews; or conference abstracts; sample size less than 20; literature that did not describe prenatal assessment or postnatal outcomes, or where the diagnosis was obtained only after birth; The CVR cutoff value of 1.6 was used to evaluate the complications caused by CLM, but not for the fetal hydrops.

### Information extraction and quality evaluation

Two researchers independently screened literature and extracted data according to the inclusion and exclusion criteria, and disagreement in the literature collection process was discussed carefully. Extracts included first author, year of publication, study type, geographic background, study sample size, number of true positive (TP) cases, number of false positive (FP) cases, number of false-negative (FN) cases, and number of true negatives (TN) cases.

The QUADAS-2 tool was applied to assess the quality of the eligible studies [18]. The QUADAS tool consists of 4 key domains that discuss patient selection, index test, reference standard, and flow of patients through the study and timing of the index tests and reference standard(flow and timing). The researcher responded "yes," "no," and "unclear" based on the content of the literature. And are phrased such that “yes” indicates a low risk of bias. The process of quality evaluation was carried out independently by two researchers, and the article's final evaluation was decided through discussion when disagreement was encountered.

### Statistical analysis

Heterogeneity due to non-threshold effects was evaluated using the Q test and heterogeneity *I*^2^. If *P* < 0.05 or *I*^2^ ≥ 50%, heterogeneity existed among the included studies, and a random-effects model was used to quantitatively pool the diagnostic accuracy of CVR; if *P* > 0.05 or *I*^2^ < 50%, homogeneity existed among the included studies, and a fixed-effects model was used to quantitatively pool the diagnostic accuracy of CVR. The calculated indexes included the pooled sensitivity, specificity, positive likelihood ratio, negative likelihood ratio, and pooled diagnostic odds ratio (DOR). Describe the receiver operating characteristic curve (ROC curve) and AUC, where the closer AUC is to 1, the higher its accurate value is. Deeks funnel chart was used to evaluate the research publication bias. Based on the Bayesian probability principle, the fagan chart represents the probability before the test, likelihood ratio, and probability after the test with three vertical axes, which is used to vividly reveal the relationship between the probability before the test, the positive likelihood ratio, the negative likelihood ratio, and the probability after the test, to evaluate the clinical diagnostic value of CVR. All analyses were performed using Stata 15.0, and a *P* value less than 0.05 was considered statistically significant.

## Results

### Literature screening results

**Fig. 1:**
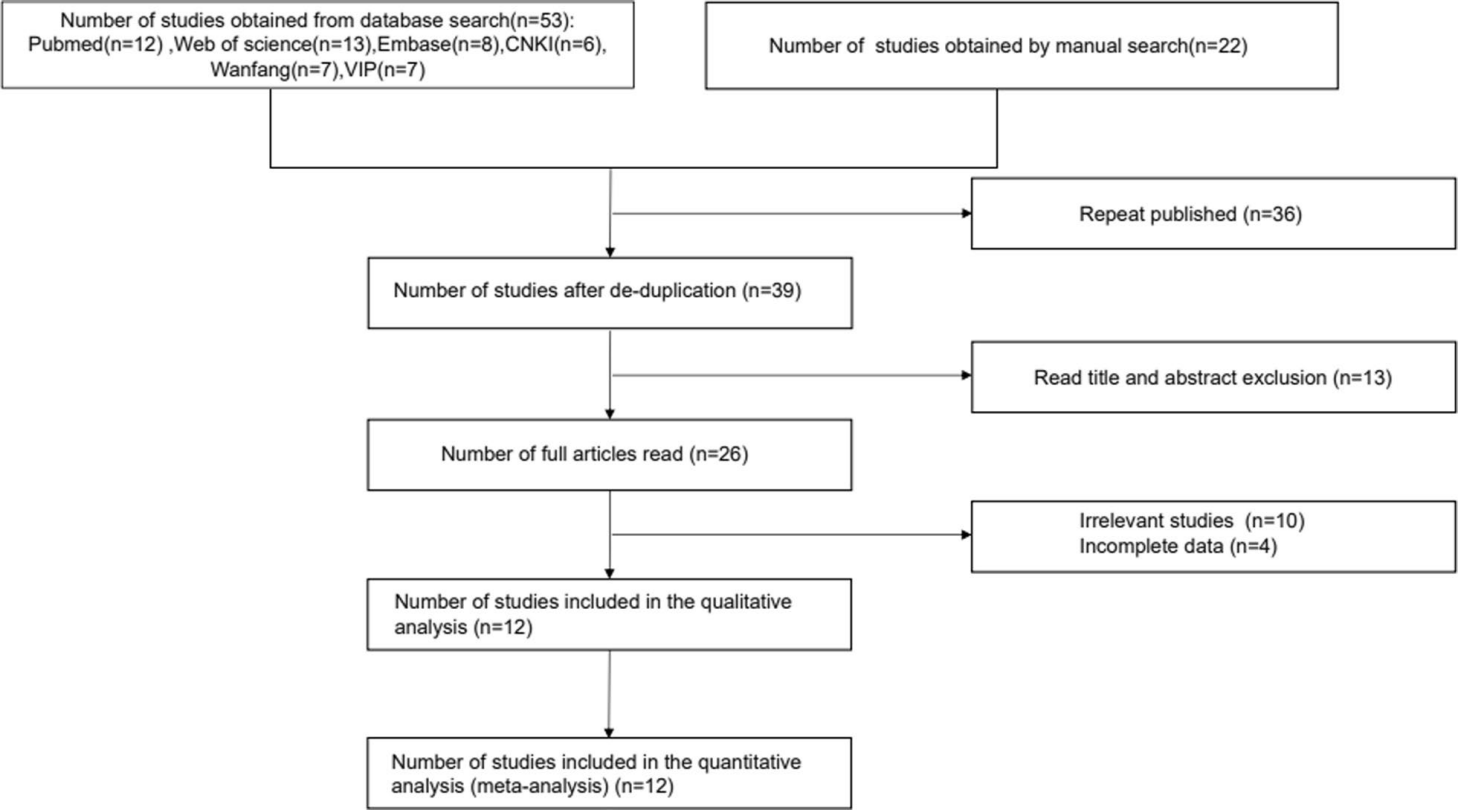
Flow diagram

The literature screening process is shown in Fig. 1. 12 articles were finally included in Meta-analysis.

### Basic information for inclusion in the literature

As shown in Table 1 The included studies were retrospective [9, 12–16, 19–23] except for one with a prospective study design [8]. Seven of the included studies were conducted in Asia [14, 16, 19–23] and four studies were conducted in North America [8, 9, 12, 13], and one study was conducted in Europe [15]. The sample size for the included studies ranged from 34–88, with a combined sample size of 815. Nine of the included studies reported a mean diagnostic gestational week; seven studies reported a mean diagnostic gestational week ≥ 24 [13–16, 20–22], two studies < 24 [9, 12], and three studies did not provide any description of the diagnostic gestational week [8, 19, 23].

**Table 1:** Basic characteristics of the 12 included literatures

Id	Author	Year	Country	Study design	Sample size	Mean diagnostic gestational week	TP	FP	FN	TN
1	Crombleholme [8]	2002	North America	Prospective	58	NR	12	4	7	35
2	Cass [9]	2011	North America	Retrospective	78	21.7 ± 4.5	11	11	1	55
3	Yong [12]	2012	North America	Retrospective	69	21	4	6	2	57
4	Ehrenberg-Buchner [13]	2013	North America	Retrospective	62	24.3	3	2	1	56
5	Zhang [14]	2014	Asia	Retrospective	68	24	7	5	0	56
6	Stoiber [15]	2017	Europe	Retrospective	34	24.2 ± 4.7	3	5	5	21
7	An [19]	2017	Asia	Retrospective	88	NR	39	5	3	41
8	Zhang [16]	2016	Asia	Retrospective	84	24	12	4	2	66
9	Chen [20]	2014	Asia	Retrospective	75	24	7	6	0	62
10	Hu [21]	2017	Asia	Retrospective	82	26.2 ± 6.5	4	7	1	70
11	Zhang [22]	2017	Asia	Retrospective	74	28	5	3	0	66
12	Ma [23]	2017	Asia	Retrospective	35	NR	3	6	0	26

*TP* true positive, *FP* false positive, *FN* false-negative, *TN* true negatives

### Study quality evaluation of the included literature

**Fig. 2:**
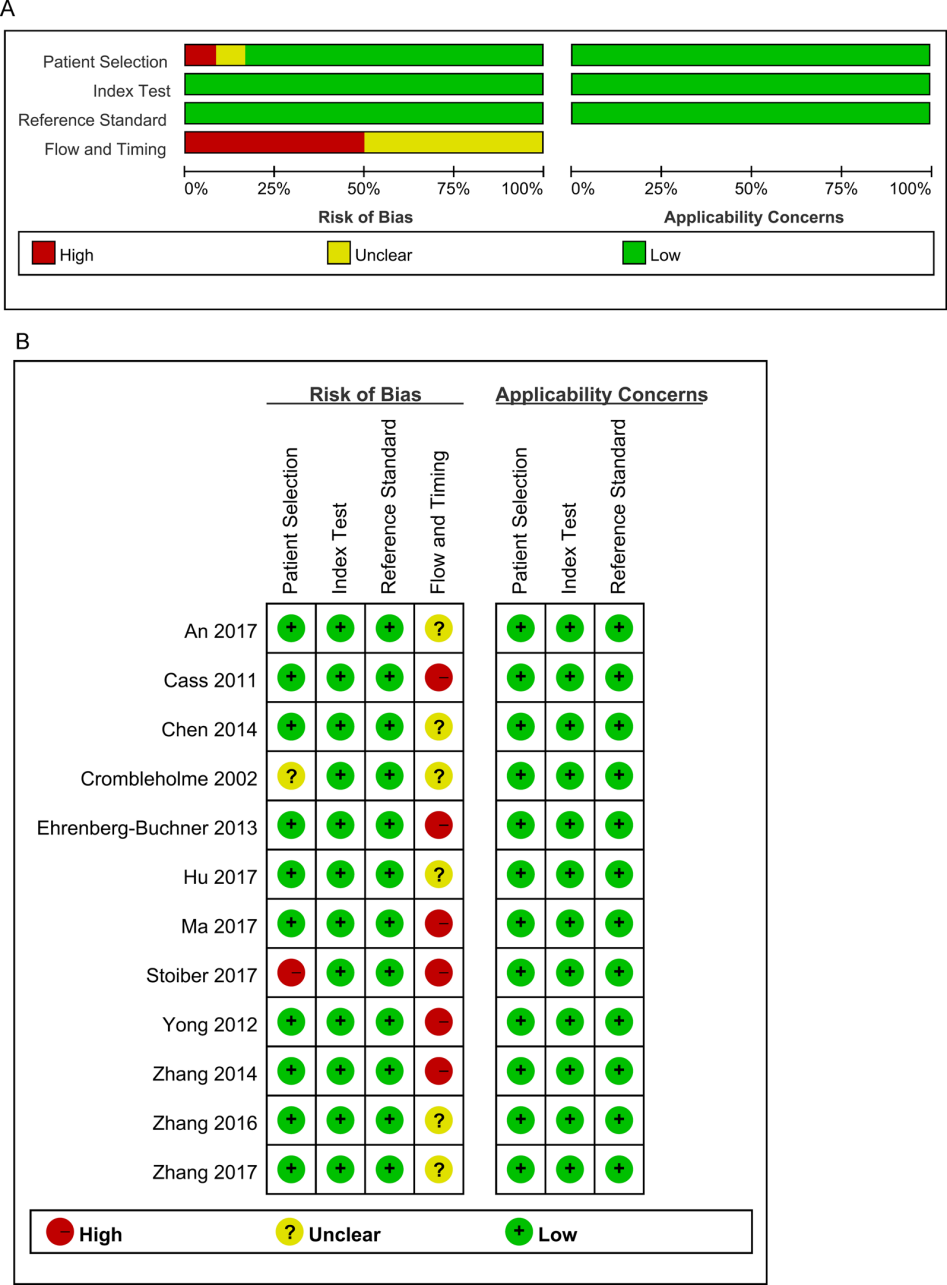
Literature quality assessment. **A** Risk of bias and applicability concerns graph. **B** Risk of bias and applicability concerns summary

The quality of the 12 articles that meet the inclusion criteria is evaluated by the QUADAS-2 tool, as shown in Fig. 2. The patient selection domain was categorized as high if the study did not avoid inappropriate exclusions. One literature [15], some patients were excluded due to multiple pregnancies and incomplete cases, and we believe that this exclusion was inappropriate, so the patient selection domain was categorized as high. Because both the reference standard and the index test were measured using objective and standard methods, the domain reference standard and the index test were categorized as low. The flow and timing domain was categorized as high if the study did not include all patients. The domain of six pieces of literature (50%) was categorized as high. Two studies [12, 13] were excluded some patients due to loss follow-up. Two studies [9, 23] excluded patients with incomplete information. And one literature [15] excluded some patients because they did not meet the inclusion–exclusion criteria. One study [14] did not explain the reasons for excluding some patients.

As for applicability, the patients were selected for continuous collection in hospitals according to relevant inclusion and exclusion methods, which we consider to have relatively high applicability. The reference standard and the index test are objective and standard methods with high applicability.

### Results of meta-analysis

#### Diagnostic accuracy

**Fig. 3:**
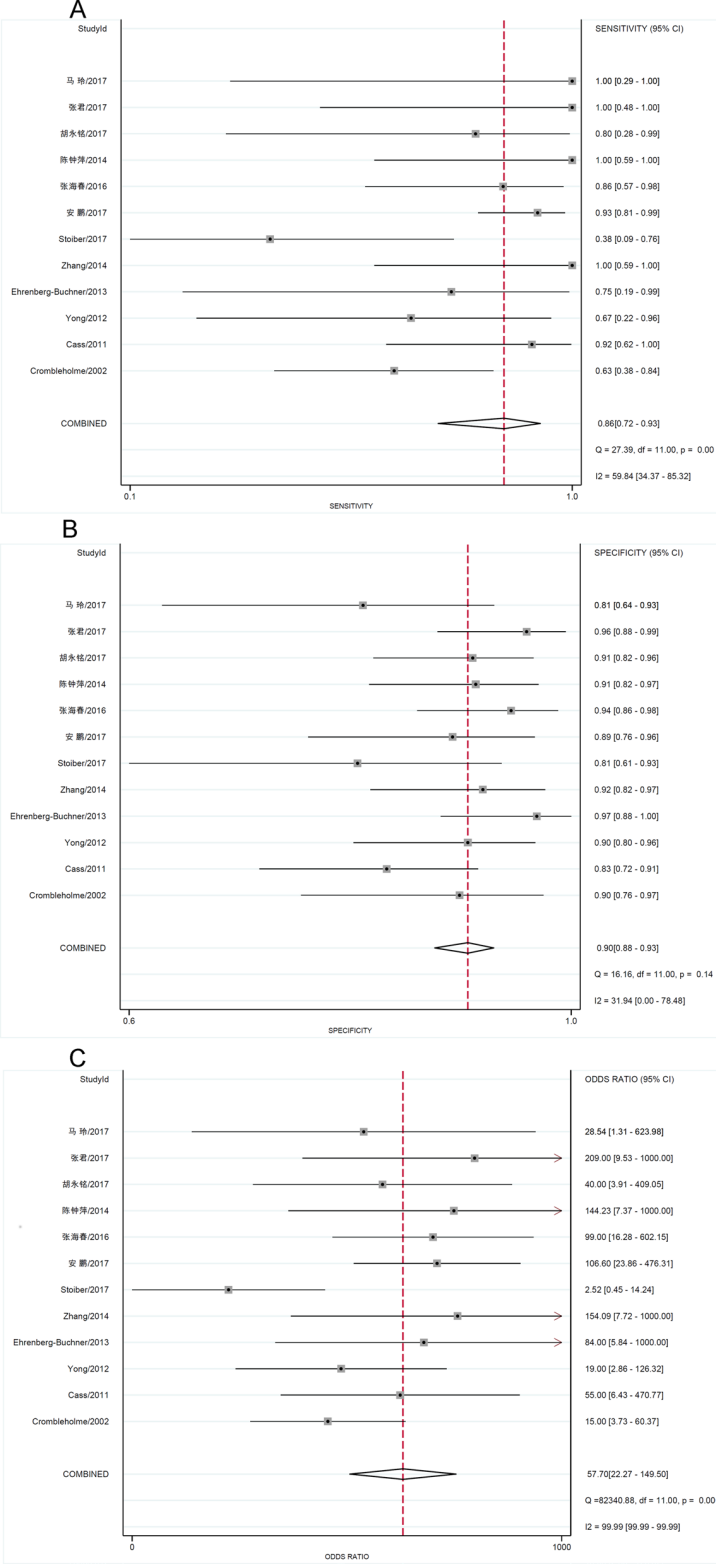
Forest plot of CVR diagnostic accuracy evaluation. **A** Pooled forest plot of CVR diagnostic sensitivity; **B** pooled forest plot of CVR diagnostic specificity degree; **C** pooled forest plot of DOR of CVR diagnosis

**Fig. 4:**
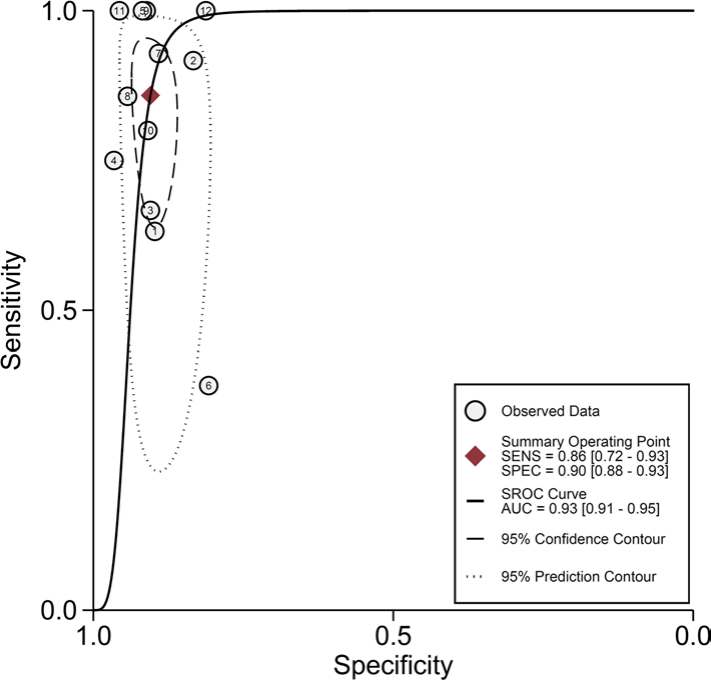
Sroc curve for CVR

We performed a diagnostic accuracy analysis of the literature that met the inclusion and exclusion criteria, as shown in Figs. 3 and 4. The pooled sensitivity and specificity of the CVR cutoff value of 1.6 were 0.86 (95% CI, 0.72–0.93; *I*^2^ = 59.84) (Fig. 3A) and 0.90 (95% CI, 0.88–0.93; *I*^2^ = 31.94) (Fig. 3B), respectively. The pooled diagnostic ratio of the CVR cutoff value of 1.6 was 58 (95% CI, 22–149; I^2^ = 100%) (Fig. 3C). The summary receiver operating characteristic (SROC) of the CVR cut off value of 1.6 was 0.93 (95% CI, 0.91–0.95) (Fig. 4).

### Subgroup analysis

**Fig. 5:**
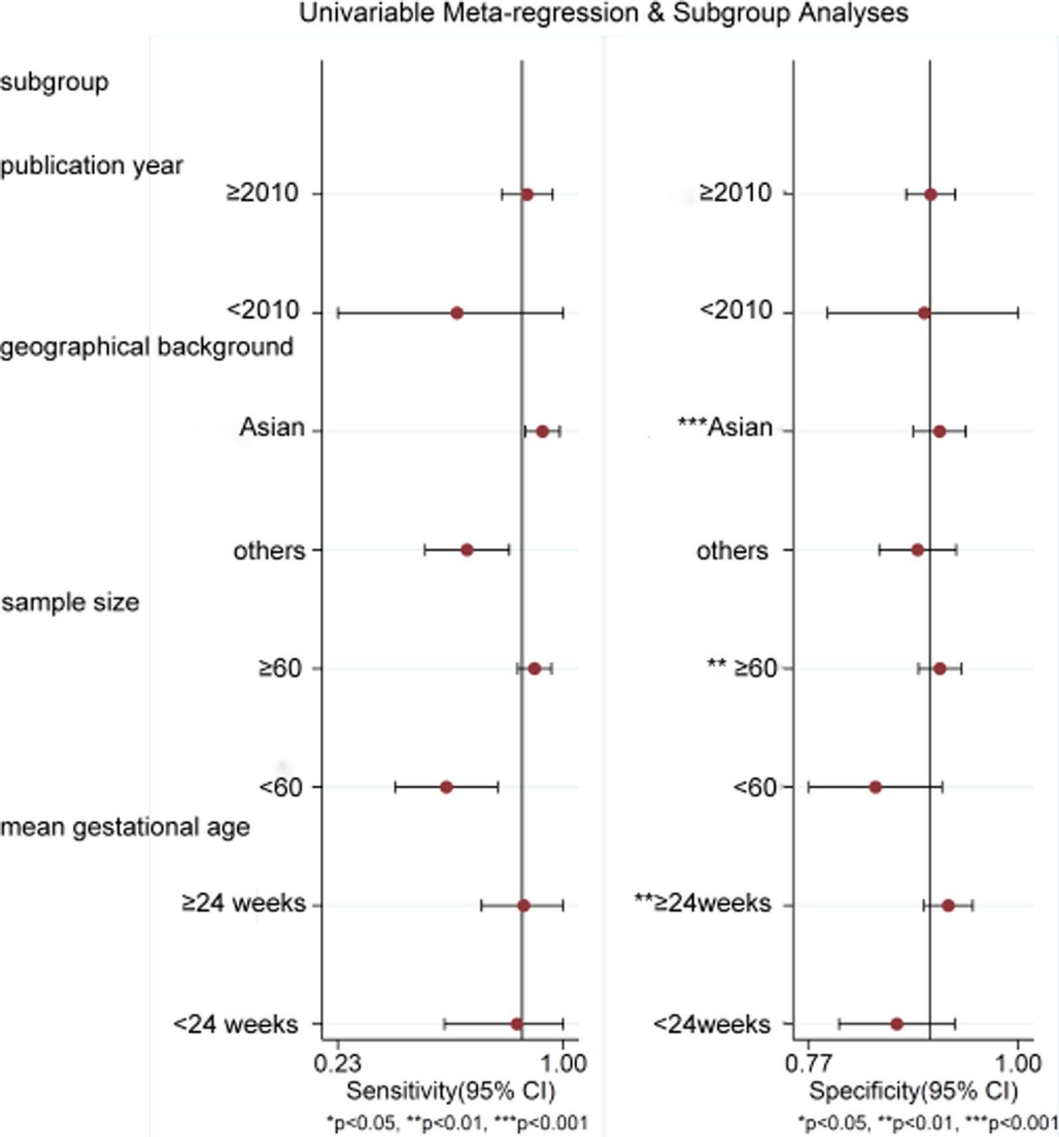
Subgroup analysis

Due to the limitation of the number of included studies, the publication year (≥ 2010, < 2010), geographical background (Asia, other), sample size (≥ 60, < 60) and mean gestational age (≥ 24 weeks, < 24 weeks) were analyzed by subgroups. The results of the study showed that the pooled specificity was significantly higher in those with a geographical background in Asia than in other regions (*P* < 0.001); the pooled specificity was significantly higher in those with a sample size ≥ 60 than in those with a sample size < 60 (*P* < 0.01); and the pooled specificity was significantly higher in those with a mean gestational age ≥ 24 weeks than in those with a diagnostic age < 24 weeks (*P* < 0.01). There was no significant difference in pooled sensitivity between subgroups and no significant difference in pooled specificity between groups in publication year (Fig. 5).

### Publication bias

**Fig. 6:**
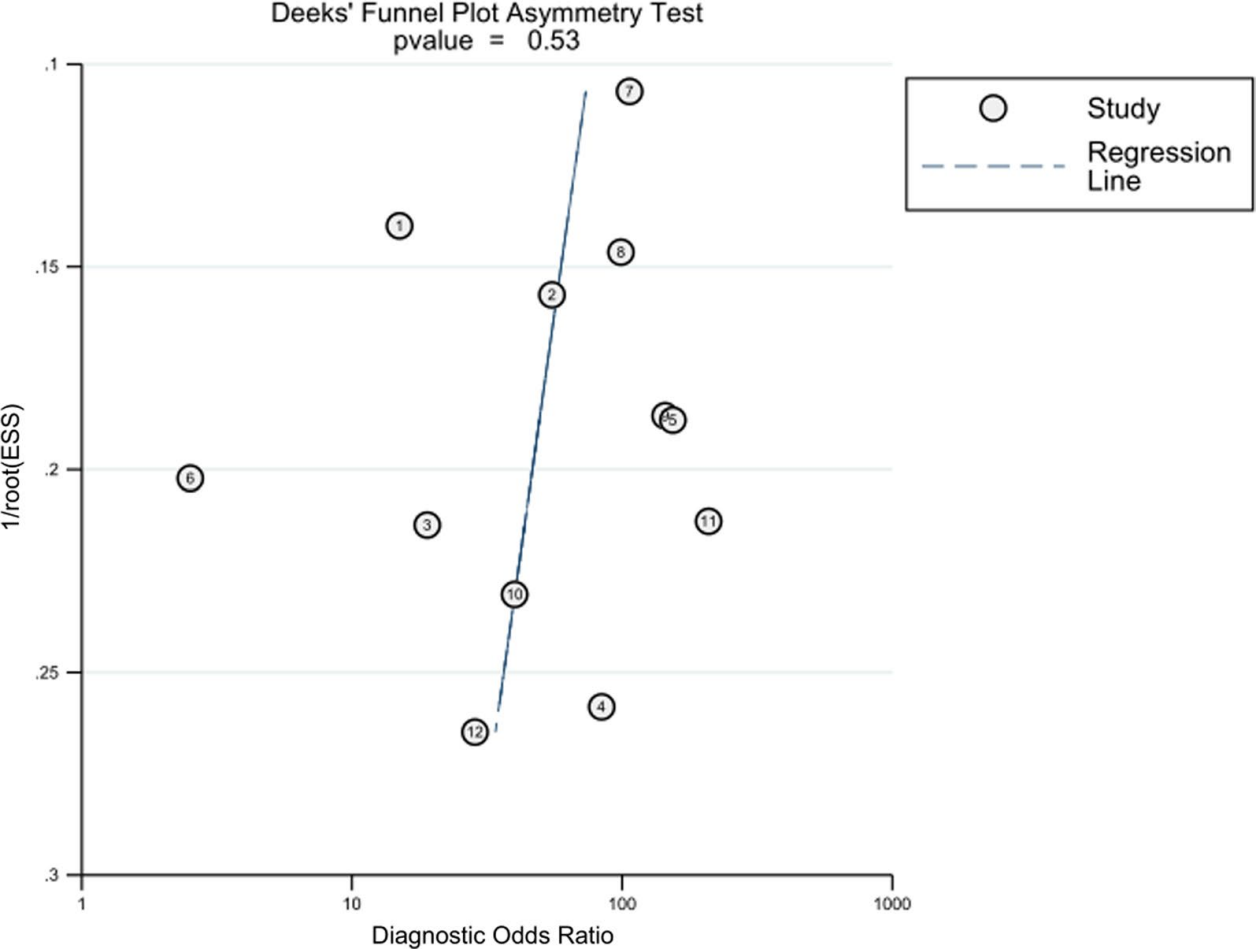
Deeks funnel chart

Deeks funnel charts were used to assess the inclusion literature of publication bias. Deeks plots showed a slope (Bias) =  − 4.87, *P* = 0.529, suggesting no significant publication bias among the included literature. This result indicates no statistically significant publication bias among the included literature (Fig. 6).

### Pre-test probability and post-test probability

**Fig. 7:**
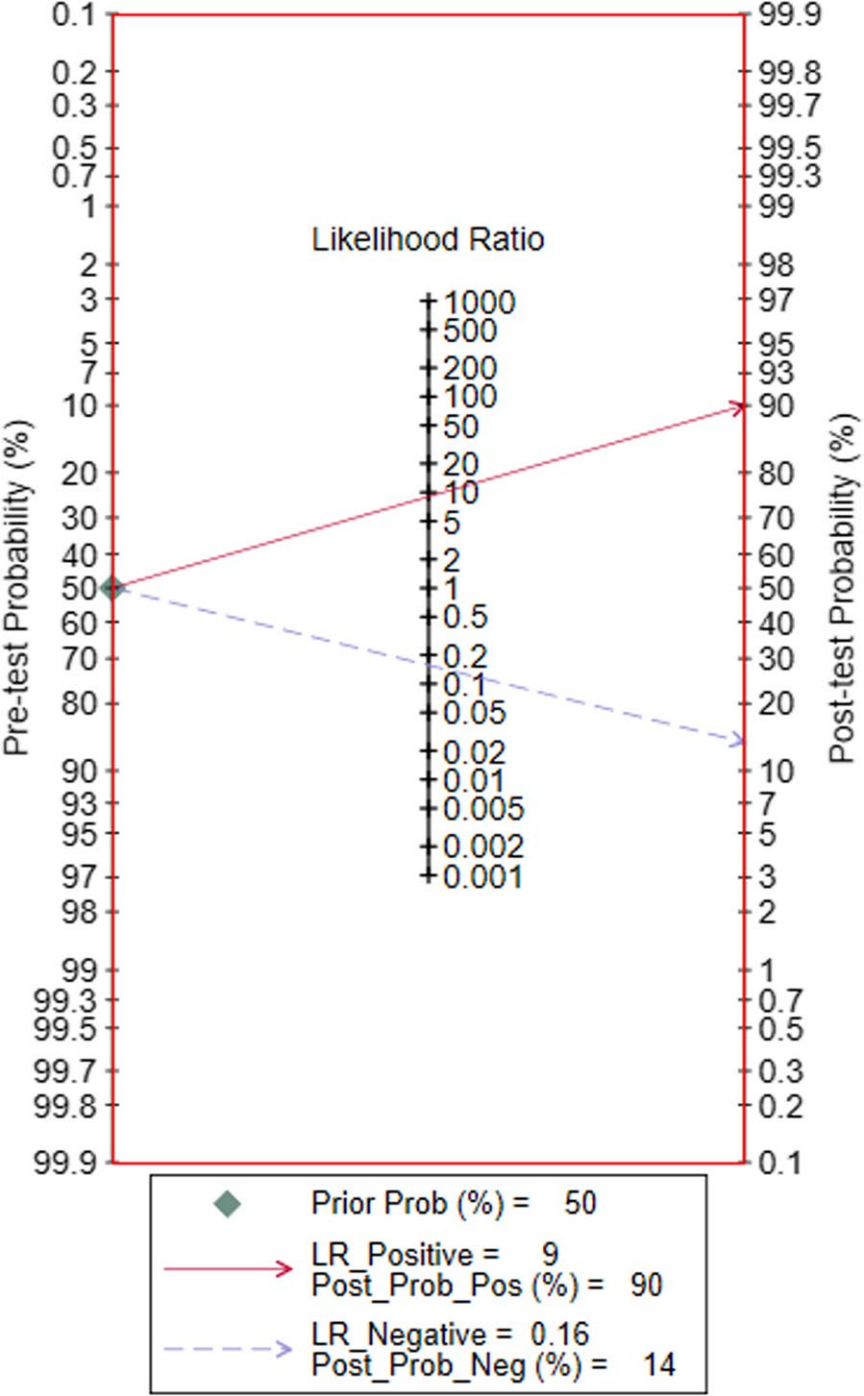
Fagan chart for clinical applicability evaluation of CVR

Fagan chart shows that with a preset probability of 50%, the probability of CLM causing fetal hydrops rises to 90% if CVR > 1.6 and decreases to 14% if CVR < 1.6 (Fig. 7).

## Discussions

Twelve studies were included in this study, and the overall quality of the included studies was high. Meta-analysis showed that the pooled sensitivity was 0.86 and the pooled specificity was 0.90. Despite the high inter-study heterogeneity, the AUC was 0.93, the diagnostic ratio was 58, and there was no publication bias, indicating a high accuracy of CVR for the diagnosis of CLM-induced fetal hydrops.

Crombleholme concluded that when the CVR was < 1.6, 86% did not have hydrops and the fetal prognosis was good; when the CVR was ≥ 1.6, 75% had hydrops and the fetal prognosis was poor [8]. The pooled sensitivity after quantitative analysis of 12 studies in this study was 0.86, and the pooled specificity was 0.90, indicating that when tested according to this method, the probability of being correctly determined as having the disease was 86%, and 14% of patients were missed; the probability of being correctly determined as not having the disease was 90%, and 10% of patients were misdiagnosed. The DOR reflects the degree of association between the results of the diagnostic test and the disease [24], and the larger the value, the better the diagnostic performance of the method [25]. The DOR in this study was 58, which reflects the good diagnostic performance of the indicator. Combining the results of all meta-analyses indicates that CVR has high accuracy in the diagnosis of CLM-induced fetal hydrops.

Fagan plot results showed that if CVR > 1.6, then CLM caused fetal hydrops to increase from a predetermined probability of 50 to 90%; if CVR < 1.6, then CLM caused fetal hydrops decrease from a predetermined probability of 50 to 14%. Despite the large inter-study heterogeneity, the AUC was 0.93 and the results of the literature quality evaluation were also good, and there was no publication bias. Therefore, the CVR cut-off value of 1.6 as an early predictor of fetal hydrops can improve the early prediction and identification of CLM-induced fetal hydrops.

The *I*^2^ values of sensitivity and the diagnostic ratio of this study were > 50%, indicating heterogeneity between studies due to non-threshold effects. The results of the subgroup analysis showed that geographic background, sample size, and mean diagnostic gestational week may be the main sources of heterogeneity. The reasons for the heterogeneity in geographic background consider that there may be differences in the detection rate of CLM due to differences in social welfare and the level of medical care, as well as different policies on abortion and legal termination of pregnancy in different countries, resulting in large differences in pregnancy outcomes in CLM [26, 27]. The average gestational week of diagnosis may be the main source of heterogeneity, considering that the lesions may change or even "disappear" as the gestational week progresses [3], which may have an impact on the occurrence and development of the later stage of the disease. Therefore, when using CVR to predict and identify fetal hydrops caused by CLM, we should consider the gestational age comprehensively.

This study also has some limitations. CLM is a rare congenital abnormality of the fetus [28], and fetal hydrops is one of its related complications. The number of studies that can meet the inclusion and exclusion criteria is small. In addition, the heterogeneity of the effect indicators included in studies is high, most of the studies are retrospective studies, and the inconsistent experience of ultrasonographers are all factors that can produce bias.

## Conclusions

To sum up, the diagnostic evaluation indexes of this study are relatively stable, and there is no publication bias. CVR has certain significance for the diagnosis of fetal hydrops caused by CLM. However, larger samples and higher-quality studies are still needed to further explore the diagnostic value of CVR1.6 in fetal hydrops caused by CLM.

## Data Availability

Not applicable.

## Abbreviations

CLM: Congenital lung malformation: CLM is a collective term for a range of disorders that include the congenital abnormalities of the lung parenchyma and its bronchovascular structures
CVR: Congenital pulmonary airway malformation volume ratio: it can be used to determine the volume ratio of the lesion to the lung, to describe the relationship between the lesion and the lung. and to predict fetal hydrops
CPAM: Congenital pulmonary airway malformation: CPAM is a nonfunctioning multicystic congenital pulmonary tissue dysarthria, with histological features of terminal bronchiolar overgrowth and the absence of normal alveoli
PS: Pulmonary sequestration: PS is a non-functional lung mass, which is separated from the normal trachea and bronchus and receives blood supply from systemic circulation
CLE: Congenital lobar emphysema: CLE is a rare developmental lung anomaly, characterized by hyperinflation of one lobe most commonly the left upper lobe
